# Subthreshold electrical stimulation as a low power electrical treatment for stroke rehabilitation

**DOI:** 10.1038/s41598-021-93354-x

**Published:** 2021-07-07

**Authors:** Kyungsoo Kim, Seung-Jun Yoo, So Yeon Kim, Taeju Lee, Sung-Ho Lim, Jae-Eun Jang, Minkyu Je, Cheil Moon, Ji-Woong Choi

**Affiliations:** 1 Brain Engineering Convergence Research Center, Daegu Gyeongbuk Institute of Science and Technology, Daegu, Korea; 2 Convergence Research Advanced Centre for Olfaction, Daegu Gyeongbuk Institute of Science and Technology, Daegu, Korea; 3 Department of Brain and Cognitive Sciences, Daegu Gyeongbuk Institute of Science and Technology, Daegu, Korea; 4 Department of Electrical Engineering, Korea Advanced Institute of Science and Technology, Daejeon, Korea; 5 Department of Information and Communication Engineering, Daegu Gyeongbuk Institute of Science and Technology, Daegu, Korea

**Keywords:** Stroke, Spike-timing-dependent plasticity

## Abstract

As a promising future treatment for stroke rehabilitation, researchers have developed direct brain stimulation to manipulate the neural excitability. However, there has been less interest in energy consumption and unexpected side effect caused by electrical stimulation to bring functional recovery for stroke rehabilitation. In this study, we propose an engineering approach with subthreshold electrical stimulation (STES) to bring functional recovery. Here, we show a low level of electrical stimulation boosted causal excitation in connected neurons and strengthened the synaptic weight in a simulation study. We found that STES with motor training enhanced functional recovery after stroke in vivo. STES was shown to induce neural reconstruction, indicated by higher neurite expression in the stimulated regions and correlated changes in behavioral performance and neural spike firing pattern during the rehabilitation process. This will reduce the energy consumption of implantable devices and the side effects caused by stimulating unwanted brain regions.

Stroke is a major brain injury that causes disabilities of body functions (e.g., movement, balance, swallowing)^1–3^. Clinically, functional recovery has been accomplished by exercise rehabilitation in the post-stroke phase and is caused by structural and functional remodeling that relies on plasticity perilesional cortex (PLC) or a remote area connected to the infarct area during the rehabilitation process^4–7^.

There have been various attempts to enhance plasticity for better functional rehabilitation using certain types of exercise^4,8–10^. In addition to exercise rehabilitation, direct electrical stimulation to the brain has been used to mediate brain activity and enhance plasticity for stroke rehabilitation^11–13^. Direct electrical stimulation has been reported to promote activity-dependent neurotrophins, which enhance axonal and dendritic sprouting^14–16^. Micro-electrophysiology systems have provided a precise approach to target neural systems for stroke rehabilitation^17,18^. Here, the region damaged by stroke is functionally replaced by an electrical device that senses neural activities and stimulates a brain region to enhance the functional connection between two regions^17^. In addition, stimulation in timing sensitive manner affect the motor rehabilitation^19^. These precise neural recording-based approaches imply that the connection between two brain regions can be reconstructed after stroke and that Hebbian plasticity can be used to strengthen the functional connection. In previous studies, the focus was on scientific reports of functional recovery by the addition of an electrical device. However, there has not been an engineering approach considering appropriate electrical stimulation power to enhance rehabilitation performance and to lower the system cost (i.e., energy consumption) and unexpected side effects. For the full implantation of an electrical treatment system, the energy consumption^20^ and side effects must be considered in addition to the functional recovery.

In the current study, we aimed to promote functional recovery with less energy after stroke. We simulated engineering possibilities to reduce energy consumption. As an engineering target, spike timing-dependent plasticity (STDP), which is a major component of Hebbian plasticity, was utilized, and we achieved synaptic weight control with less energy. Using the knowledge acquired from the simulation study, we experimented with the engineering scenario in vivo and observed functional recovery with smaller electrical stimulation energy.

## Results

### Subthreshold electrical stimulation for causal activation

**Figure 1:**
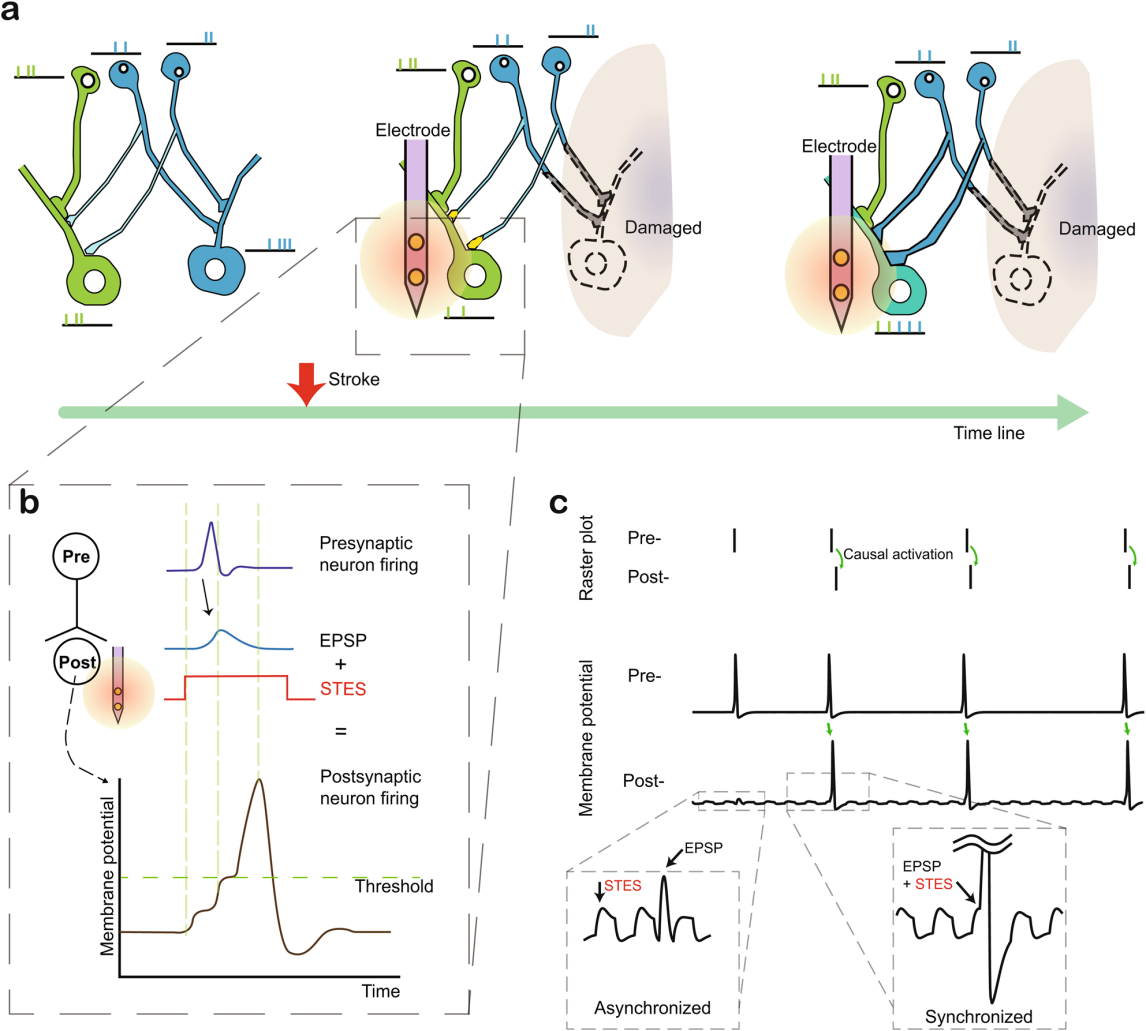
Synaptic remapping by electrical stimulation after stroke. (**a**) The connection between pre- and postsynaptic neurons are rebuilt by stroke and electrical stimulation. After stroke newly made connections are strengthened by electrical stimulation. (**b**) Incoming EPSP from presynaptic neurons and additional potential provided by STES induces a postsynaptic action potential following the presynaptic action potential. (**c**) The upper graph, raster plot, displays the spike timing at pre- and postsynaptic neuron (Pre- and Post- respectively). The bottom graph is the simulation result of two connected neurons with STES. When STES and presynaptic activation occur asynchronously, postsynaptic neurons do not fire (left-highlighted square). When presynaptic firing and STES occur together, postsynaptic fire followed presynaptic neuron activation (right highlighted square).

In PLC after stroke, the population of gained and lost synapses is increased^21^ for functional remapping. Our engineering purpose was to promote the survival rate of newly made synaptic connections after stroke. While synaptic remapping was boosted with the gain and loss of synaptic connections^22^, we strengthened the synaptic weight to prevent synaptic connection loss. Based on this distinct mechanism of synaptic remapping after stroke (Fig. 1a), we studied the ability of STDP to control synaptic weight by manipulating synaptic activation. In STDP, the activation between pre- and postsynaptic neurons must be causal. To manipulate the synaptic weight using STDP, we proposed the application of subthreshold electrical stimulation (STES) of the population of postsynaptic neurons simultaneous to physical exercise to more strongly activate the population of presynaptic neurons (Fig. 1b). STES cannot make the membrane potential reach the action potential threshold. STES alone is a small bias. When there is additional potential from presynaptic neuron, the membrane potential reaches the action potential to fire the neuron affected by STES. This process naturally makes causal activation between pre- and postsynaptic neurons. This causal activation is hypothesized to result in functional recovery based on the synaptic remapping process.

Using the two engineering points on the pre- and postsynaptic sides, we examined causal activation in silico (Fig. 1c). Since it is difficult to measure the quantitative weight change in synapse and the condition of pre- and postsynaptic neurons, we examined the initial idea of STES in computer simulation. When stimulation was asynchronized, even though the presynaptic neuron fired and caused an excitatory postsynaptic potential (EPSP), the postsynaptic neuron did not fire an action potential. Since neither presynaptic input nor STES alone were able to initiate an action potential, the presynaptic neuron only fired while the postsynaptic neuron was silent in the raster plot. On the other hand, when presynaptic input and STES occurred simultaneously, the overall membrane potential reached the action potential threshold, resulting in firing of the postsynaptic neuron. As a result, the raster plot shows that sequential causal activation resulted in postsynaptic firing following presynaptic firing. These asynchronous and synchronous cases are possible cases that can occur with STES.

### Controlling synaptic weight by STES with less energy

We explored the changes in synaptic weight caused by engineering in pre- and postsynaptic neurons. Synaptic weight is regulated by the STDP mechanism in the simulation since STDP is one of the major properties of Hebbian plasticity^23^. We utilized the STDP model proposed by Song et al.^23^ (for details see the Methods section). The STDP mechanism senses the firing timing difference between pre- and postsynaptic neurons and updates the weight as Δ*w*. If there is postsynaptic firing following presynaptic firing, Δ*w* is positive, and the synaptic connection is strengthened. On the other hand, if postsynaptic firing leads to presynaptic firing, the synaptic weight degrades, and the synaptic connection is weakened.

**Figure 2:**
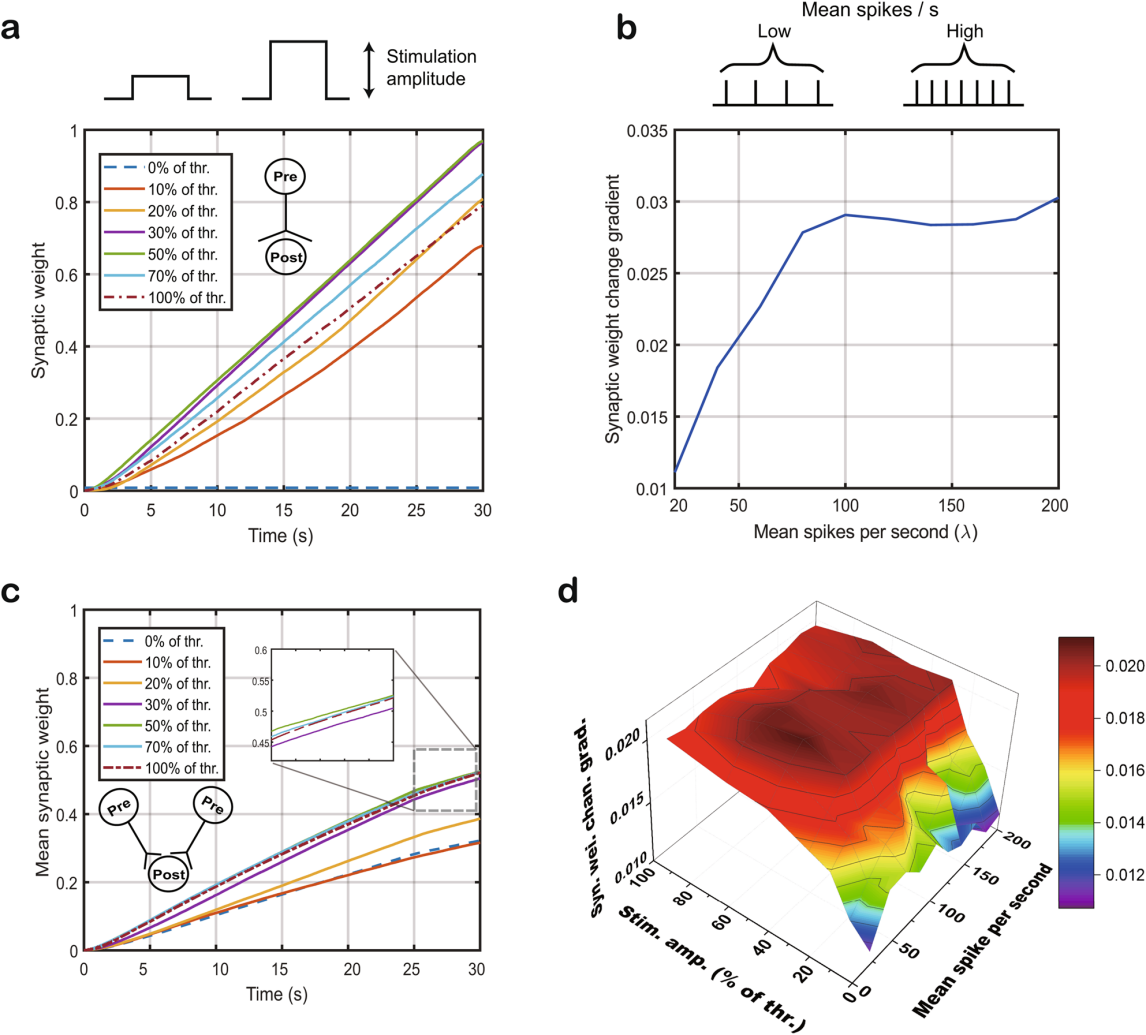
Synaptic weight change induced by STES and presynaptic firing. (**a**) Changes in synaptic weight in response to STES at various amplitudes from 0 to 100% of the action potential threshold amplitude. (**b**) The firing rate was controlled by mean spikes per second (i.e., λ in the Poisson process), from 10 to 200 when 30% threshold was chosen for the simulation. (**c**) Two presynaptic neurons are connected to one postsynaptic neuron to test synaptic competition effect by STES. The mean synaptic weight of two synapses increased in the simulation with presynaptic competition based on STDP. (**d**) The synaptic weight change gradient was calculated by the stimulation amplitude and the presynaptic firing rate (mean spikes per second) in the simulation with presynaptic neuron competition based on STDP.

To focus on synaptic weight change by pre- and postsynaptic excitation, we designed one presynaptic neuron connected to one postsynaptic neuron. A Poisson random process was applied for presynaptic neurons to fire randomly following the mean spikes per second (i.e., λ in the Poisson process). For the postsynaptic neuron, electrical stimulation was applied from 0 (no stimulation) to 100% of the threshold level (i.e., the minimum electrical stimulation level needed to cause an action potential). As shown in Fig. 2a, electrical stimulation at levels other than 0% (i.e., no stimulation) increased the synaptic weight. Notably, although STES (i.e., 0% < stimulation level < 100%) increased the synaptic weight, STES alone did not result in postsynaptic neuron firing due to the lack of the synchronous occurrence of the action potential in the presynaptic neuron and the electrical stimulation in the postsynaptic neuron. Since the presynaptic neuron fires randomly, the ratio of the causal activation depends on the mean spikes in the Poisson process. As the mean spike rate increased, the gradient of the change in the synaptic weight increased (Fig. 2b). At higher mean spike rates, the synaptic weight is more frequently increased, indicating that higher activation in the presynaptic side results in a stronger connection between neurons.

Since the presence of just one presynaptic connection to one postsynaptic neuron is not physiologically representative, we added one more presynaptic neuron to add synaptic competition^23–25^. The activities of the two presynaptic neurons followed the Poisson process with the same mean spike rate but were designed to have an independent random process. The mean synaptic weight was measured by averaging the two synaptic weights. The results in Fig. 2c show that the gradients of each electrical stimulation level were less differentiated than those shown in the simulation with one presynaptic neuron (Fig. 2a). The electrical stimulation of more than 30% of the threshold level showed small performance variance comparing to the other levels (i.e., 0% = stimulation level ≤ 20%) (Fig. 2c). The 30% of the threshold level performed near the best that 50% of the threshold level did. Note that 30% of the threshold level took 60% current level of the best performed current level but only have 3.5% weight difference at 30 s. The overall gradient was halved because synaptic competition. Interestingly, the no-simulation case (i.e., 0% of stimulation level) showed presence in synaptic weight change due to the accumulation of the synaptic input potentials from the two presynaptic neurons. We explored the parameters of stimulation amplitude level and mean spike rate to obtain the best combination (Fig. 2d). The overall pattern of the gradient of synaptic weight change shows that there was small performance variance with stimulation amplitudes over 30% having less than 5% performance difference to the best combination at 50% of stimulation amplitude and 60 of mean spike level, implying that we may be able to reduce the electrical power for functional recovery after stroke. A low level of electrical stimulation has distinct advantages of saving power consumption. For example, a 30% stimulation amplitude reduces the power consumption to 9%, potentially saving power for implantable devices by combining presynaptic excitation and STES on the postsynaptic side.

### Combination of a motor task and STES for stroke rehabilitation

**Figure 3:**
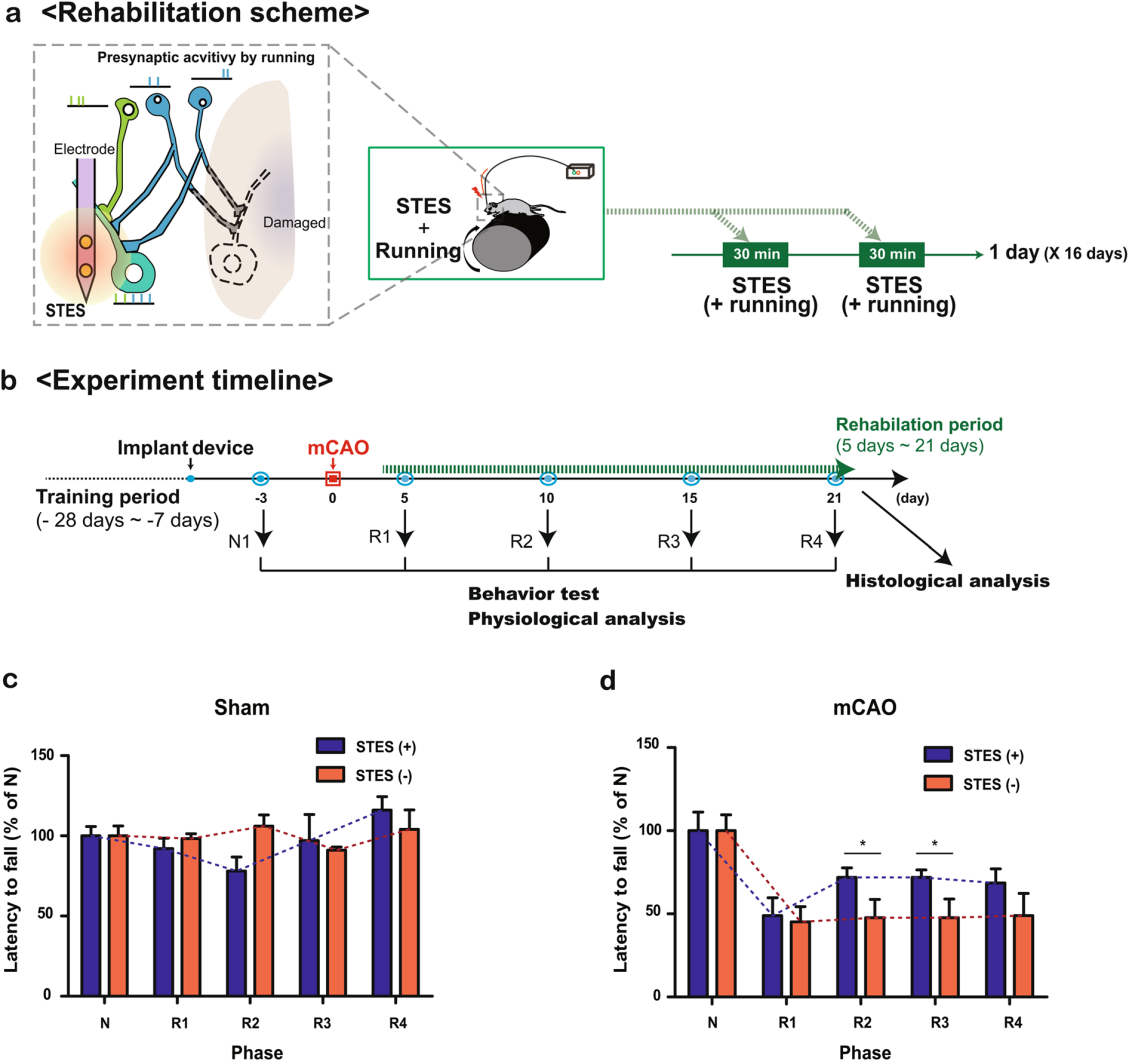
Overall experimental scheme and behavioral test analysis. (**a**) Rehabilitation scheme: STES was matched with rotarod training. The STES began at post-mCAO day 3 ~ 4, and the stimulation started at the same time as the running task for 30 min twice a day for 16 days. (**b**) Schematic of the rehabilitation timeline: the timeline shows the training period, mCAO, and rehabilitation period. After the training period (-28 days ~ -7 days), the device was implanted. Then, the pre-mCAO behavior and spike units were collected (N). mCAO surgery was performed at day 0, and the rehabilitation process (5 days ~ 21 days) began simultaneously with the collection of the post-mCAO behavior and spike units at post-mCAO day 5 (R1 ~ R4). After the rehabilitation process, all of the rats were sacrificed for histological analysis. Analysis of the latency to fall from the rotarod of (**c**) the sham (two-way ANOVA; interaction, ns; group, ns; phase, ns) and (**d**) mCAO animals with or without STES (two-way ANOVA; interaction, ns; group, *p < 0. 05, F(1,70) = 11.1; phase, ***p < 0.0001, F(4,70) = 17.62). More than 4 animals were monitored for each condition (n = 4, sham STES(-); n = 4, sham STES( +); n = 5, mCAO STES(−) and n = 8, mCAO STES( +)).

To validate the combination of presynaptic activation and STES on the postsynaptic side, we tested the engineering scenario described above the simulation in vivo. We combined a running task and micro electrical stimulation with STES to enhance the functional connection between the motor function and plasticity change in vivo. In order to induce presynaptic side activation, repeated rotarod running was performed; at the same time, STES was utilized to manipulate postsynaptic activity (Fig. 3a). This treatment process was performed twice a day with 30 trials per session beginning at poststroke day 3 or 4 and lasting for 16 days. We started poststroke training as early as we could to enhance the plasticity effect targeting critical period after stroke when spine turnover is maximized^21^. The rotarod running task was chosen as the presynaptic activation stimulus to result in broad activation in the brain, and STES was performed in the right-hemispheric motor areas (M1). The performance of which during the preparatory period of a finger movement with the dominant right hand would be affected by stroke stress (Fig. S1a). The motor threshold (MT), which is the lowest injected current level necessary to force motor movement (Fig. S1c), was defined by vertical multipolar electrical stimulation (Fig. S1b) with 20–50 μA, which is approximately half of the current level reported in previous studies^17,26,27^. In addition, we applied STES by applying electrical stimulation with 30% of the MT (i.e., 6–15 μA). To ensure the proper level of STES, we defined the MT at three distinct time points throughout the rehabilitation period. During the experiment, the change in the MT was less than 5 μA (Fig. S1d). We performed electromagnetic (EM) field analysis to estimate the area effectively stimulated by the STES. We modeled the virtual electrode and placed it in the rat phantom model, which mimics a real rat from an electrical propagation point of view (Fig. S2a-c). The electrical field (E-field) vector result in Fig. S2b shows that the multipolar stimulation with the vertical electrode formation resulted in a vertical current in cortex layer 5. Potential changes of approximately 100 and 30 mV were observed at 50 and 100 μm away from the STES location, respectively, in the simulation (Fig. S2d). This result suggests that the STES affects an area less than 100 μm from the stimulation site, which may minimize the stimulation of nontargeted brain areas.

Reversible ischemia (occlusion-reperfusion) was induced to make the transient middle cerebral artery occlusion (mCAO) model because middle cerebral artery failure is the most common type of ischemic stroke^28^. Then, we used four groups of trained rats with implanted electrodes: sham non-STES (STES(-)), sham STES( +), mCAO STES(-), and mCAO STES( +). To examine the details during the rehabilitation process, we performed certain analyses using a particular rehabilitation timeline (Fig. 3b). During the rehabilitation process following mCAO, behavioral and physiological analyses were simultaneously started at post-mCAO days 3 ~ 4. Rehabilitation with behavioral and physiological analyses continued until post-mCAO day 21. Baseline behavior and physiology levels were evaluated at phase N (before mCAO), and changes post mCAO were evaluated at phase R1 (less than 5 days after mCAO), R2 (6 to 10 days after mCAO), R3 (10 to 15 days after mCAO), and R4 (more than 15 days after mCAO).

### STES with a motor task enhances motor function recovery

To address the effect of combining motor training with STES on motor function recovery, we evaluated the behavioral performance of mCAO rats performing motor running (i.e., rotarod running test). Rotarod running is regarded as the most sensitive and efficient method to detect motor impairment after brain injury^29–31^. For the behavioral performance analysis, the latency to fall from the rotarod was measured. Rats in the mCAO STES( +) condition exhibited improvement in their motor function compared to those in the mCAO STES(-) condition (two-way ANOVA; interaction, ns; group, *p < 0. 05, F(1,70) = 11.1; phase, ***p < 0.0001, F(4,70) = 17.62). Especially in phase R2 (mCAO STES(-), 45.1 ± 20.2; mCAO STES( +), 71.9 ± 10.6) and R3 (mCAO STES(-), 47.6 ± 20.7; mCAO STES( +), 71.9 ± 8.3) there are significant difference between the groups (unpaired two-tailed t-test; R2, *p < 0.05, t = 2.81, df = 14; R3, *p < 0.05, t = 2.89, df = 14) and the improvement was maintained throughout the rehabilitation process (Fig. 3d). Interestingly, rats in the sham STES condition did not show alterations or improvements in their behavioral performance compared to those in the sham STES(-) condition (Fig. 3c; two-way ANOVA; interaction, ns; group, ns; phase, ns), suggesting that the effect of STES in mCAO rats is dependent on the mCAO environment. These data indicate that the enhanced motor performance resulting from STES plus motor training is accomplished by long-lasting M1 neuronal stimulation only under conditions of mCAO.

### Functional recovery relies on dendritic connections

**Figure 4:**
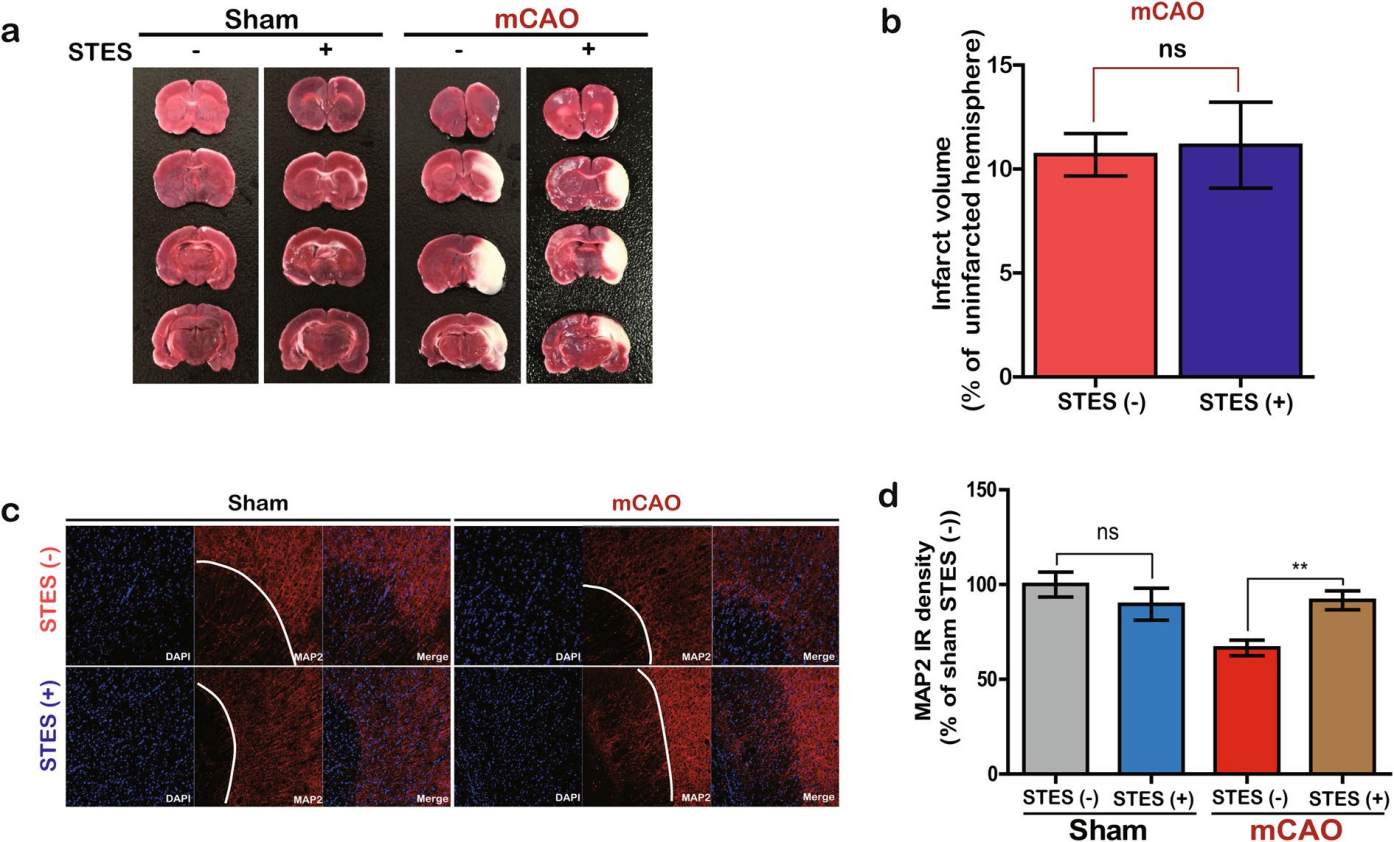
Infarct volume and histological analysis. (**a**) TTC staining of the brains in each group. Nonischemic areas appear red, and ischemic areas appear white. (**b**) Quantitative analysis of the cerebral infarct volume in each mCAO group (mCAO STES( +) and mCAO STES(-)). The bar graph reflects the infarct volume in each group. Neurological deficits were determined by stereological analysis with the ImageJ program. (**c**) Representative images of the primary motor cortex, M1, in each group. Tissues were immunostained using MAP2 antibodies (red) and visualized under a confocal microscope. MAP2 immunoreactivity (IR) across all the conditions showed different signal intensities. (**d**) Stereological analysis of the MAP2 IR intensity of the M1 cortex region with the ImageJ analysis program. All data are presented as the mean ± standard error of the mean (SEM). For the statistical analysis, unpaired two-tailed t-test was performed with the Prism software (GraphPad software, USA), ** *P* < 0.005 and ns *P* > 0.05 denote statistical significance.

We confirmed whether the infarct size was affected by the STES. Examination of the brains after all experimental procedures were completed. We found that mCAO induced an extensive infarction throughout all ipsilateral brain regions, including the cerebral cortical and subcortical areas, but not in contralateral brain regions (Fig. 4a), confirming that mCAO induced brain damage. Importantly, mCAO STES( +) did not decrease the size of the infarction area in the ipsilateral brain regions, and there were no significant differences in the size of the infarction area between the mCAO STES( +) and mCAO STES(-) conditions (STES(-), 195.9 ± 17.43; STES( +), 204.1 ± 17.29 (mm^3^)) (Fig. 4b).

Previous studies have clearly shown that functional remapping is mainly accomplished by neural plasticity in the target region^32^. The neuron-specific protein microtubule-associated protein 2 (MAP2) is the most widely used marker of dendritic regions of neurons. Thus, the immunohistological intensity of MAP2 was used to represent neural plasticity. To examine changes in plasticity under the conditions tested here, we monitored MAP2 expression in the target regions. mCAO clearly resulted in decreased MAP2 expression. Furthermore, STES significantly upregulated MAP2 expression in the target regions of the mCAO rats (mCAO STES( +), 91.7 ± 14.1; mCAO STES(−), 66.5 ± 11.67; unpaired two-tailed t-test, *p* < 0.005, t = 3.627, df = 14) to levels similar to those observed in the sham STES condition (Fig. 4c, d; sham STES( +), 89.6 ± 23.9). Taken together, these results suggest that STES failed to prevent total structural damage from the infarction in the brain after mCAO but did modulate the neural plasticity that may promote recovery of motor function.

### Functional rebuilding by STES with a motor task

**Figure 5:**
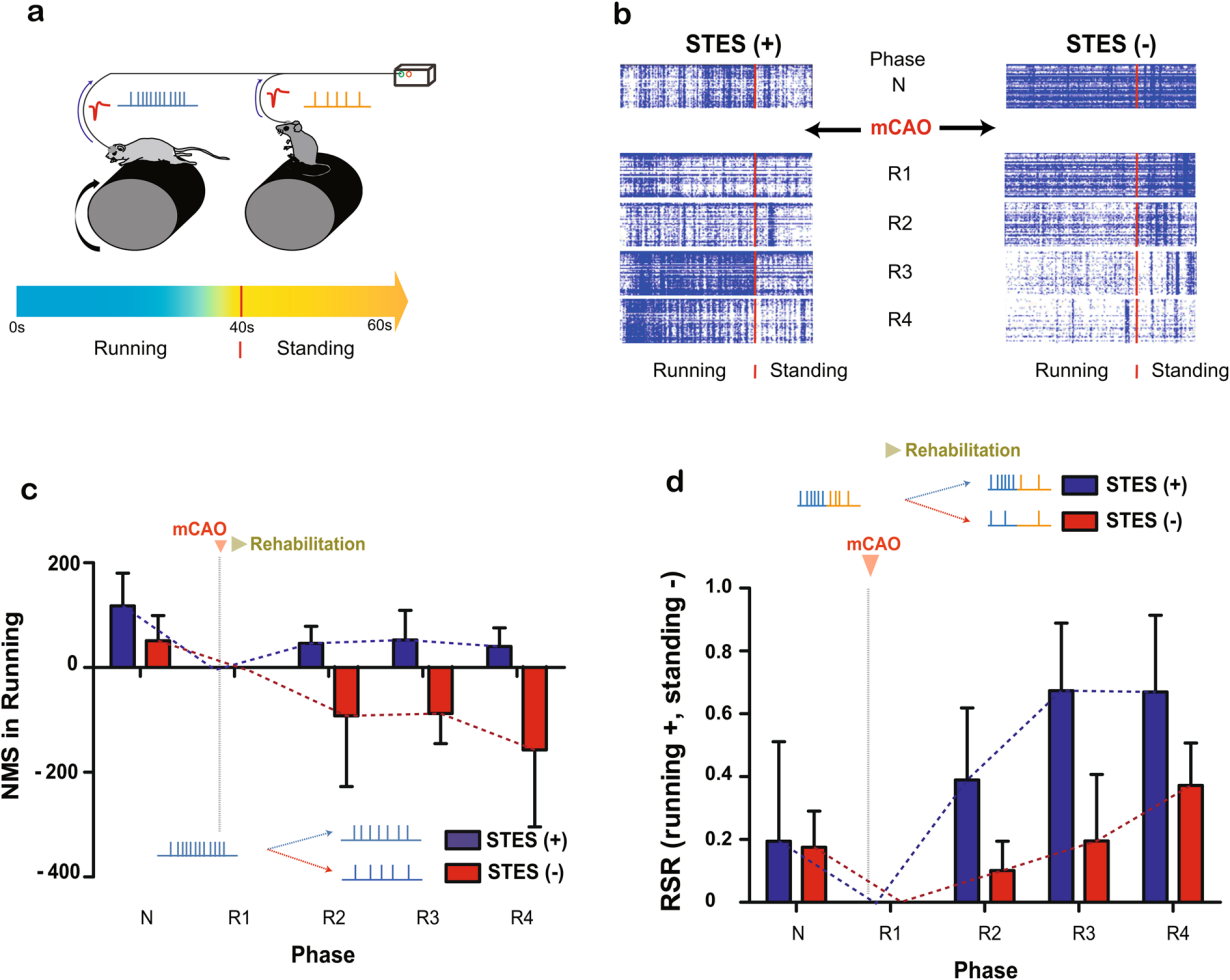
Physiological analysis. (**a**) The neural spike signal was measured while a rat was running and standing for 40 and 20 s, respectively. (**b**) The spike firing in the mCAO STES( +) and mCAO STES(-) groups is shown in the daily raster plot for each phase (N, R1, R2, R3, and R4; pre mCAO, less than 5 days, 10 days, 15 days after the mCAO, and more than 15 days after mCAO, respectively). (**c**) The NMS in the running state; the NMS for mCAO STES( +) group does not change, while the NMS for the mCAO STES(−) group decreases after mCAO (two-way ANOVA; interaction, ns; group, *p < 0.05, F(4,40) = 6.9). (**d**) The RSR on the rotarod; neural firing activation for the mCAO STES( +) group was focused more on the running task after the mCAO (two-way ANOVA; interaction, ns; group, *p < 0.05, F(1,44) = 5.22; phase, *p < 0.05, F(4,44) = 2.9).

To address changes in neural plasticity by combining motor training and STES in the mCAO group, multi-unit spike firing was recorded and analyzed during stroke and the rehabilitation process. As shown in Fig. 5a, multi-unit recording was performed at STES training to measure neural firing during running and standing, representing a trained and nontrained motor task, respectively. Note that the stimulation and recording were performed separately to avoid recording contamination by electrical stimulation artifacts. The detected spikes in the raster plot show different tendencies during the stroke and rehabilitation processes (N ~ R4) (Fig. 5b). In each phase, there was an increase in spike population density during rehabilitation in the mCAO STES( +) rats, while the spike population in mCAO STES(-) rats was sparser than in mCAO STES( +) rats after R1.

For quantitative analysis of the changes in neural plasticity, the number of multi-unit spikes (NMS) was measured. The NMS represents the number of spikes fired in the running state compared to that at baseline in the R1 phase when rehabilitation began (Fig. 5c), with the NMS at R1 set to 0. Both the mCAO STES(-) and mCAO STES( +) rats exhibited a decrease in the NMS between N (mCAO STES( +), 117.8 ± 152.4; mCAO STES(-), 51.1 ± 96.6) and R1(baseline) in the running state (Fig. 5c), suggesting that the neurons near the electrodes fired less due to the loss of synaptic connections caused by mCAO. After mCAO, the neural activity continued to decrease in the mCAO STES(-) (Linear regression; slope = -46.8, *p* = 0.3), even after rehabilitation. In contrast, the mCAO STES( +) recovered neural firing after R1, showing an increase in NMS (Linear regression; slope = 12.7, *p* = 0.4). This result shows that STES with rotarod running promoted neural activity after mCAO.

To compare the spike firing tendency between the trained motor task (running) and the untrained motor task (standing), the spike firing ratio between the standing and running state (RSR) was monitored. The RSR represents a ratio indicating a normalized NMS between the running and standing state (for technical details see the Methods section) (Fig. 5d). The RSR for both groups showed that the neural activity was focused on the trained motor task after R1 in the mCAO STES( +). Interestingly, there was significant difference in RSR between mCAO STES( +) and mCAO STES(-) group (two-way ANOVA – group: *p* = 0.02, F(1,44) = 5.22, phase: *p* = 0.03, F(4,44) = 2.9). This suggests that the population of synaptic connections related to the trained motor task survived more than the other functional connections, resulting in a firing pattern biased to the trained motor task. This functional rebuilding is highlighted in the correlation analysis between the behavioral test results and spike firing patterns. There was a highly correlated rehabilitation tendency between motor function recovery and neural plasticity change with a 0.99 normalized cross-correlation coefficient (the maximum is 1) between the behavioral test and spike firing rate change in the mCAO STES( +), comparted to a 0.77 normalized cross-correlation coefficient in the mCAO STES(-).

## Discussion

### Engineering plasticity for stroke rehabilitation

Our engineering approach appeared to generate behaviorally relevant circuits through changes in neural plasticity. According to a previous study, sporadic diffuse reconnection may provide some level of long-term innate recovery from stroke except in directly damaged regions of the brain^4^. Specifically, Hebbian plasticity plays a key role in making novel connections in undamaged brain regions especially in PLC^33,34^. Electrical stimulation has been reported to upregulate MAP2 expression in undamaged brain regions, implying that stimulation changes the neural plasticity during rehabilitation. Along with the histological observation, neural spike firing was also upregulated when STES was combined with motor training (Fig. 5c). Taken together, our results suggest that combining STES with motor training may lead to motor function recovery associated with changes in neural plasticity even without structural reconstruction in the infarct area.

In the rehabilitation process, brain stimulation can improve recovery from stroke by elaborating plasticity, in the form of gaining or losing synaptic connections^33^. This plasticity involves simultaneous pre- and postsynaptic activation related to the repeated training^21^. Because synaptic plasticity is dependent on the spike timing of pre- and postsynaptic neurons, the rehabilitation protocol must be designed to ensure that the postsynaptic neurons fire immediately after the presynaptic neurons. To create specific circumstances, we adopted a combination of two distinct and independent variables in our rehabilitation scheme. One was the existing concept of rehabilitation upregulating innate plasticity in undamaged brain regions within the critical period after stroke^21^. The other was adopting STES combined with motor training to upregulate and focus plasticity in an extrinsic manner. Importantly, the repeated motor training and STES were applied simultaneously. The STES simulation study suggested that the low stimulation level (30% of the MT) caused more bias in the trained motor task, increasing the background potential around the electrodes, potentially resulting in causal excitation between presynaptic neurons that are afferent neurons to the M1 cortex and neurons in the M1 cortex. Using STDP, our engineering approach provides an environment encouraging the strengthening of synaptic connections rather than the loss of connections. Therefore, functional recovery was accomplished by increasing the survival of neural connections during dendritic remapping after stroke.

Our physiological results from the multi-unit spike firing analysis suggest that connections between brain regions may be strengthened by STES with motor training early in rehabilitation (R1 ~ R2), together with a dramatic improvement in behavioral performance in this period. During this early critical rehabilitation stage, a certain range of circuits related to the trained motor task may be increased and enhanced. After R2, there was no significant increase in the number of spikes; similarly, the enhancement in behavioral performance also stalled. Because the nervous system has a certain range of recovery capacities to change the properties of its circuits^35–37^, this constraint of plasticity may be a physiological limitation. The most interesting part of our rehabilitation process was R2 ~ R3, immediately following R1 ~ R2. Although the R2 ~ R4 period showed a similar tendency in the behavioral performance and spike firing results, the tendency for the spike signal to be focused toward motor training constantly increased during this specific period of the rehabilitation process (R2 ~ R3), indicating that a certain range of circuits related to the trained motor task was specifically reinforced during the late critical rehabilitation stage.

In clinical use of electrical stimulation for stroke rehabilitation, invasiveness, stimulation timing, intensity, and task specificity are considered^38^. Real-time closed loop stimulation control which requires recording, complex processing, and higher hardware specs is utilized for the stimulation timing optimization^39,40^. Our result shows that low intensity stimulation during task can bring more co-firing (Fig. 1c) without any control. This implies that STES approach can loosen the timing control constraint over closed loop approach. STES is also a benefit for invasiveness. Since less invasive approach needs higher stimulation power to deliver enough stimulation effect, this causes wider stimulation in the brain. The less power requirement in STES can benefit for resolving this issue. To take the benefit in clinical use, as the results in Fig. 2a,b imply, delivering effective intensity level to the target brain region needs to be studied in various invasiveness conditions based on STES and the patient’s condition to perform a task bringing more brain activity.

### Reducing power consumption and side effects

As implanted electronic systems require durable systems in a small package^20^, battery size is an important matter for system design. Compared to sensing and monitoring devices, electrical stimulation devices consume a relatively large amount of power, and the effort is limited by the development of a low-impedance electrode^41,42^. In the current study, performing STES based on the STDP mechanism provided the capability of reducing the energy consumption for electrical stimulation to achieve functional recovery. Even with just 9% ((30% stimulation amplitude)^2^ impedance) of the stimulation power necessary to result in neural excitation, our engineering approach achieved functional recovery after stroke. This concept enables engineering approaches to reduce power consumption in electrical stimulation, not only improving electrode manufacturing techniques but also informing future systematic approaches to physiological engineering.

Although direct electrical stimulation has the benefit of manipulating neural activation and promoting functional recovery after stroke, the technique is limited by the production of undesired stimulation effects (e.g., motor and speech problems)^43,44^. The side effects are mainly caused by the stimulation of a large population of cells, including unwanted neurons around the stimulation site. In order to reduce the side effects, optogenetic stimulation has been applied in animal experiments. However, optogenetic tools cannot be the solution in clinical application because their use in the clinic is prohibited^45^. STES may be an alternative solution to avoid side effects. As the amplitude of the electrical stimulation decreases, the size of the affected area shrinks exponentially, reducing the exposure of untargeted brain regions to electrical stimulation. Therefore, STES provides the advantage of reducing power consumption as well as avoiding side effects.

## Methods

### Neuron and STDP model

In order to examine the effects of STDP, a simple neuron model was adopted for simulation. A neuron was modeled with two compartments, cell body and dendrite. The dendrite part contained an embedded passive channel for current leaking with a conductance of 0.001 S/cm^2^ and a reversal potential of 65 mV. For the cell body, the Hodgkin-Huxley mechanism was included to simulate the active process of neurons. Sodium, potassium and leak conductance were set at 0.12 S/cm^2^, 0.036 S/cm^2^, and 0.0003 S/cm^2^, respectively, and a reversal potential of -54.3 mV was applied. The neurons were isolated so as not to be affected by the electrical propagation from the other. One synapse was built to monitor the synaptic modification caused by STDP between the two neurons. The basal activity of neurons was modeled by a Poisson random process, and in the presynaptic neuron, event-related activation was added following a Poisson random process with λ.

For the STDP model in the current study, the STDP model proposed by Song et al.^23^ was employed. The model provides a symmetric and asymmetric form of STDP and updates the synaptic weight as follows:

1 $$\Delta w = \left\{ {\begin{array}{*{20}c} {w_{{\max }} A_{ + } \exp [(t_{{pre}} - t_{{post}} )/\tau _{ + } ]\,\,\,\,\,\,\,if\,\,t_{{pre}} < t_{{post}} } \\ { - w_{{\max }} A_{ - } \exp [(t_{{pre}} - t_{{post}} )/\tau _{ - } ]\,\,\,\,\,\,\,if\,\,t_{{pre}} \ge t_{{post}} } \\ \end{array} } \right.$$

where *w*_max_ is the maximum weight, *A*_+_ and *A*_-_ are the step size for weight increase and decrease, respectively, *t*_pre_ and *t*_post_ are the spike timing in the pre- and postsynaptic neuron, and τ_+_ and τ_-_ are the time constant, respectively. In the current study, *A*_-_τ_-_/*A*_+_τ_+_ = 1.06 as in Song et al.^23^.

The simulation was performed by the NEURON simulator (version: 7.5) under a python environment (version: 2.7). Current was utilized as the source type of the electrical stimulation. Electrical stimulation directly injected current into the cell body membrane and modulated the membrane potential. The level of the current amplitude, stimulation timing and time duration were controlled.

### Experimental cerebral ischemic stroke

We induced reversible ischemia (occlusion-reperfusion) to make the mCAO model. Briefly, 30 animals were randomly divided into experimental (n = 16 [electrical stimulation: 8; electrical nonstimulation: 8]) or sham (n = 14 [electrical stimulation: 8; electrical nonstimulation: 6]) groups. Under anesthesia by isoflurane inhalation in medical-grade oxygen, their sternocleidomastoid muscle was dissected, and the right common carotid artery (CCA), external carotid artery (ECA), and internal carotid artery (ICA) were exposed. The arteries were carefully separated from the surrounding vagus nerves and connective tissue. ECA and CCA was tied, and a filament was then inserted from the ECA to the ICA and advanced to the MCA^46^. After 2 h of occlusion, the animals were reanesthetized, and the filament was gently withdrawn to allow reperfusion of the ischemic region.

### Quantification of the stroke volume

The Animal Care and Use Committee of the Daegu Gyeongbuk Institute of Science and Technology (DGIST) approved all animal protocols. Also, this study was carried out in compliance with the ARRIVE guidelines. After all rehabilitation procedures, the rats were euthanized under anesthesia after reperfusion, and the brains were rapidly removed. Coronal sections were cut into 2-mm-thick slices and stained with standard 2% TTC for 20 min at 37°C^17,18^. The pale-appearing infarcted areas and the uninfarcted areas were digitally analyzed using the NIH ImageJ software. The area of infarction from each slide was summed and presented as a percentage of the volume of the uninfarcted hemisphere.

### Behavioral motor testing

All animals were housed in individual cages. Before induction of ischemia, all animals received training in the rotarod test (20 rpm) for 14 days. The rotarod test was used to examine balance and coordination^44–46^. The rotating drum was accelerated from 0 to 50 rpm over 3 min, and the latency in seconds for the animals to fall off the drum was recorded. Each session included three consecutive trials with a maximum time of 600 s, and the mean fall latency was calculated from three trials.

### Histology and immunohistochemistry

Animals were anesthetized by intraperitoneal injection of 65 mg/kg ketamine with 5 mg/kg xylazine. The rats were then transcardially perfused with prechilled phosphate-buffered saline (PBS, pH 7.6)^47^. The heads were removed, skinned, and postfixed overnight in 4% paraformaldehyde in PBS at 4°C^48^. The mandibles were discarded, and the trimmed heads were skinned and fixed by immersion in the same fixative for 1 week at 4 °C. The heads were decalcified in 10% EDTA (pH 7.0) for 1 week at 4 °C. After decalcification, the specimens were washed, dehydrated in increasing concentrations of ethanol, and transferred into xylene for tissue clearing. The specimens were infiltrated with Paraplast and embedded. Frontal sections (coronal, 6 μm) were cut serially from the tip of the nose to the posterior extension of the cortex layer, and each section was preserved on MAS-coated slides (Matsunami Glass, Japan).

### Electrode implantation and electrical stimulation

A single shank electrode (A1 × 16-3 mm-100–703, NeuroNexus) was placed at the primary motor cortex (M1, AP =  + 1 mm, ML =  + 2 mm, DV = -2 mm) on the gray matter in the brain, and the reference electrode was located on the skull. The implanted electrode had a sixteen-channel vector array with a 1.5-mm-long recording area for which the recording sites were evenly distributed. A repeated pattern of [E, G] was applied to the electrode, for which E is the electrical stimulation site (active site) and G is the ground site. A biphasic pulse with 50 Hz of stimulation frequency and 30% of the MT was applied. The anodic duration and cathodic duration were 200 μs, and the gap between them was 40 μs.

### Spike detection and sorting

Intracortical recording was performed with the Tucker-Davis Technologies (TDT) neurophysiology system (RZ2, TDT) with a 24-kHz sampling frequency using the implanted 16-channel single shank electrode. The signals were filtered by a high pass filter at 800–3000 Hz. After filtering, threshold-based spike detection was performed by taking the spikes above the threshold. The threshold was set by calculating four times the standard deviation of the signal recorded for all the recording days as

1 $$h^{c} = 4\sqrt {\frac{1}{{DN}}\sum\limits_{{d = 1}}^{D} {\sum\limits_{{n = 1}}^{N} {\left| {x_{d}^{c} (n) - \mu _{d}^{c} } \right|^{2} } } }$$

where $x_{d}^{c} (n)$ is the recorded data at channel $c = [1,2, \cdots ,16]$ on day $d = [ - 3, - 2, - 1,3, \cdots ,D]$ with the data point $n = [1,2, \cdots ,N]$, and $\mu$ is the mean calculated as

2 $$\mu _{d}^{c} = \frac{1}{N}\sum\limits_{{n = 1}}^{N} {x_{d}^{c} (n)}$$

Note that the threshold is determined by long-term (i.e., *D* days) measurement data instead of one-day measurement data.

Spike sorting was performed after spike detection. Three principal components were used for the features, and K-means clustering with k = 9 sorted the spikes. The spike firing time was measured for each sorted spike and marked as a dot in the raster plot in Fig. 5b.

### The NMS, RSR, and normalized cross correlation

The number of spikes detected in the running and standing state at phases N ~ R4 was counted. The NMS was calculated by counting the spikes in each phase and then subtracting the number of spikes fired at R1 as a baseline:

3 $$NMS_{{state}} (p) = E\{ K_{{d \in p}}^{{state}} \} - E\{ K_{{d \in R1}}^{{state}} \}$$

where $E\{ \,\,\}$ is the expectation operator, and $K_{{d \in p}}^{{state}}$ is the counted spike firing at the days in the phase $p = [N,R1,R2,R3,R4]$ in the state $state = [{\text{running}},\text{standing} ]$.

The RSR was calculated to evaluate whether the spike firing pattern was biased to the running or standing state. The number of daily firing spikes in the running and standing state was normalized by dividing the total number of spikes in each day. Because the running state was recorded two times longer than the standing state, in the normalization, the spike number was multiplied by different weights (i.e., 1 for the running state and 2 for the standing state). Then, the normalized spike firing was compared, and the baseline (i.e., normalized spike firing at R1) was subtracted as

4 $$RSR(p) = mean\left( {\frac{{K_{{d \in p}}^{{{\text{running}}}} - 2K_{{d \in p}}^{{{\text{standing}}}} }}{{K_{{d \in p}}^{{{\text{running}}}} + 2K_{{d \in p}}^{{{\text{standing}}}} }}} \right) - mean\left( {\frac{{K_{{d \in R1}}^{{{\text{running}}}} - 2K_{{d \in R1}}^{{{\text{standing}}}} }}{{K_{{d \in R1}}^{{{\text{running}}}} + 2K_{{d \in R1}}^{{{\text{standing}}}} }}} \right)$$

The mean of the NMS $NMS_{{running}} (p)$ and the behavior motor test result $M(p)$ were compared by normalized cross correlation. The data range in both graphs was rescaled (i.e., min = 0 and max = 1). Then, the normalized cross-correlation coefficient was calculated as

5 $$NXCORR = \frac{{E\{ \overline{{NMS}} _{{running}} (p)\bar{M}(p)\} }}{{\sigma _{{\overline{{NMS}} _{{running}} }} \sigma _{{\bar{M}}} }}$$

where $\overline{{NMS}} _{{running}}$ and $\bar{M}$ are the rescaled NMS and behavioral motor test results, respectively, and $\sigma$ is the standard deviation.

### Electromagnetic (EM) simulation with a rat phantom

EM simulation was performed with the Sim4life (ver. 2.2, SPEAG) software platform. The 3D electrode model was implanted into the brain of a small male rat phantom model (198 g, Sprague Dawley) (Fig. S1a). The EM low frequency solver (EM LF) was used to estimate the physics of the EM propagation with Maxwell’s equation. The vector of the electric field and the electrical potential were estimated with a quasi-static approximation.

### Statistical analysis

Unpaired two-tailed t-test was used to measure the significance of the differences between two conditions. Multiple groups were compared using ordinary two-way analysis of variance (ANOVA) with Tukey test. F with degree of freedom is in F(DFn, DFd). As a posthoc comparison, the criterion for statistical significance was set at P < 0.05. Statistical analysis and linear regression were performed with GraphPad Prism (ver. 5 and 9).

## Supplementary Information

Supplementary Information 1.

Supplementary Information 2.

Supplementary Information 3.

Supplementary Information 4.

Supplementary Information 5.

## References

[1] Kothari R (1997). Patients' awareness of stroke signs, symptoms, and risk factors. Stroke J. Cerebral Circulat..

[2] Mandelzweig L, Goldbourt U, Boyko V, Tanne D (2006). Perceptual, social, and behavioral factors associated with delays in seeking medical care in patients with symptoms of acute stroke. Stroke J. Cereb. Circulat..

[3] Williams LS, Bruno A, Rouch D, Marriott DJ (1997). Stroke patients' knowledge of stroke. Influence on time to presentation. Stroke J. Cereb. Circulat..

[4] Murphy TH, Corbett D (2009). Plasticity during stroke recovery: from synapse to behaviour. Nat. Rev. Neurosci..

[5] Carmichael ST (2003). Plasticity of cortical projections after stroke. Neuroscientist.

[6] Nudo RJ (2007). Postinfarct cortical plasticity and behavioral recovery. Stroke J. Cereb. Circulat..

[7] Calautti C (2010). The relationship between motor deficit and primary motor cortex hemispheric activation balance after stroke: longitudinal fMRI study. J. Neurol. Neurosurg. Psychiatry.

[8] Lang CE (2009). Observation of amounts of movement practice provided during stroke rehabilitation. Arch. Phys. Med. Rehabil..

[9] Langhorne P, Bernhardt J, Kwakkel G (2011). Stroke rehabilitation. Lancet.

[10] Macko RF (2005). Treadmill exercise rehabilitation improves ambulatory function and cardiovascular fitness in patients with chronic stroke: a randomized, controlled trial. Stroke J. Cereb. Circulat..

[11] Adkins-Muir DL, Jones TA (2003). Cortical electrical stimulation combined with rehabilitative training: enhanced functional recovery and dendritic plasticity following focal cortical ischemia in rats. Neurol. Res..

[12] Cheng X (2012). Cortical electrical stimulation with varied low frequencies promotes functional recovery and brain remodeling in a rat model of ischemia. Brain Res. Bull..

[13] Baba T (2009). Electrical stimulation of the cerebral cortex exerts antiapoptotic, angiogenic, and anti-inflammatory effects in ischemic stroke rats through phosphoinositide 3-kinase/Akt signaling pathway. Stroke J. Cereb. Circulat..

[14] Zhu W (2011). Intranasal nerve growth factor enhances striatal neurogenesis in adult rats with focal cerebral ischemia. Drug Delivery.

[15] Markus A, Patel TD, Snider WD (2002). Neurotrophic factors and axonal growth. Curr. Opin. Neurobiol..

[16] Schinder AF, Poo M-M (2000). The neurotrophin hypothesis for synaptic plasticity. Trends Neurosci..

[17] Guggenmos DJ (2013). Restoration of function after brain damage using a neural prosthesis. Proc. Natl. Acad. Sci..

[18] Gulati T (2015). Robust neuroprosthetic control from the stroke perilesional cortex. J. Neurosci..

[19] Ramanathan DS (2018). Low-frequency cortical activity is a neuromodulatory target that tracks recovery after stroke. Nat. Med..

[20] Rao S, Chiao J-C (2015). Body electric: Wireless power transfer for implant applications. IEEE Microwave Mag..

[21] Brown CE, Aminoltejari K, Erb H, Winship IR, Murphy TH (2009). In vivo voltage-sensitive dye imaging in adult mice reveals that somatosensory maps lost to stroke are replaced over weeks by new structural and functional circuits with prolonged modes of activation within both the peri-infarct zone and distant sites. J. Neurosci..

[22] Kamikubo Y (2006). Long-lasting synaptic loss after repeated induction of LTD: independence to the means of LTD induction. Eur. J. Neurosci..

[23] Song S, Miller KD, Abbott LF (2000). Competitive Hebbian learning through spike-timing-dependent synaptic plasticity. Nat. Neurosci..

[24] Fiete IR, Senn W, Wang CZ, Hahnloser RH (2010). Spike-time-dependent plasticity and heterosynaptic competition organize networks to produce long scale-free sequences of neural activity. Neuron.

[25] Song S, Abbott LF (2001). Cortical development and remapping through spike timing-dependent plasticity. Neuron.

[26] Burnett MG (2006). Electrical forepaw stimulation during reversible forebrain ischemia decreases infarct volume. Stroke J. Cereb. Circulat..

[27] Chen B (2015). Electro-acupuncture exerts beneficial effects against cerebral ischemia and promotes the proliferation of neural progenitor cells in the cortical peri-infarct area through the Wnt/beta-catenin signaling pathway. Int. J. Mol. Med..

[28] Aspey BS, Cohen S, Patel Y, Terruli M, Harrison MJ (1998). Middle cerebral artery occlusion in the rat: consistent protocol for a model of stroke. Neuropathol. Appl. Neurobiol..

[29] Dimyan MA, Cohen LG (2011). Neuroplasticity in the context of motor rehabilitation after stroke. Nat. Rev. Neurol..

[30] Bei W (2007). Neuroprotective effects of a standardized extract of Diospyros kaki leaves on MCAO transient focal cerebral ischemic rats and cultured neurons injured by glutamate or hypoxia. Planta Med..

[31] Takeuchi N, Izumi S (2012). Noninvasive brain stimulation for motor recovery after stroke: mechanisms and future views. Stroke Res. Treatment.

[32] Nudo RJ (2013). Recovery after brain injury: mechanisms and principles. Front. Hum. Neurosci..

[33] Nudo RJ, Wise BM, SiFuentes F, Milliken GW (1996). Neural substrates for the effects of rehabilitative training on motor recovery after ischemic infarct. Science.

[34] Brown CE, Wong C, Murphy TH (2008). Rapid morphologic plasticity of peri-infarct dendritic spines after focal ischemic stroke. Stroke J. Cereb. Circulat..

[35] Soleman S, Yip PK, Duricki DA, Moon LD (2012). Delayed treatment with chondroitinase ABC promotes sensorimotor recovery and plasticity after stroke in aged rats. Brain J. Neurol..

[36] Nagahara AH, Tuszynski MH (2011). Potential therapeutic uses of BDNF in neurological and psychiatric disorders. Nat. Rev. Drug Discov..

[37] Ehrenreich H (2009). Recombinant human erythropoietin in the treatment of acute ischemic stroke. Stroke J. Cereb. Circulat..

[38] Bao S-C, Khan A, Song R, Tong RK-Y (2020). Rewiring the lesioned brain: electrical stimulation for post-stroke motor restoration. J. Stroke.

[39] Parastarfeizabadi M, Sillitoe RV, Kouzani AZ (2020). Multi-disease deep brain stimulation. IEEE Access.

[40] Zhou A (2019). A wireless and artefact-free 128-channel neuromodulation device for closed-loop stimulation and recording in non-human primates. Nature Biomed. Eng..

[41] Deng M (2011). Electrochemical deposition of polypyrrole/graphene oxide composite on microelectrodes towards tuning the electrochemical properties of neural probes. Sens. Actuat. B Chem..

[42] Zhou H, Li T, Duan YY (2012). Reduce impedance of intracortical iridium oxide microelectrodes by hydrogel coatings. Sens. Actuat. B Chem..

[43] Blomstedt P, Hariz MI (2006). Are complications less common in deep brain stimulation than in ablative procedures for movement disorders?. Stereotact. Funct. Neurosurg..

[44] Mandat T, Hurwitz T, Honey C (2006). Hypomania as an adverse effect of subthalamic nucleus stimulation: report of two cases. Acta Neurochir..

[45] Cheng MY (2014). Optogenetic neuronal stimulation promotes functional recovery after stroke. Proc. Natl. Acad. Sci. U.S.A..

[46] Lee S (2014). Middle cerebral artery occlusion methods in rat versus mouse models of transient focal cerebral ischemic stroke. Neural Regen. Res..

