# Supplementary figures and images for: Subthreshold electrical stimulation as a low power electrical treatment for stroke rehabilitation
Source: Sci Rep. 2021 Jul 7;11:14048. doi: 10.1038/s41598-021-93354-x (PMC8263745; doi:10.1038/s41598-021-93354-x)

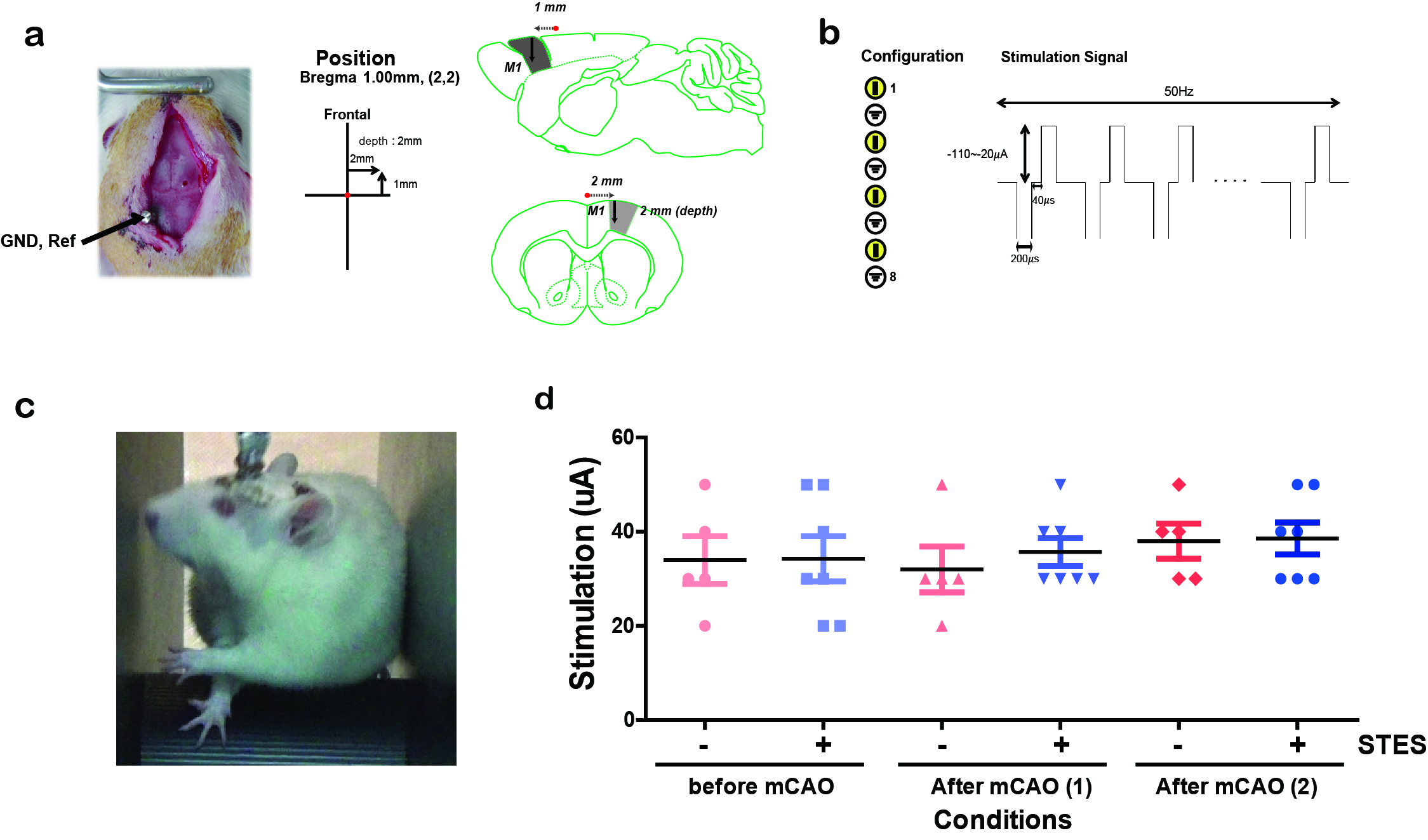

Supplement: Supplementary file 2 — Supplementary Information 2. [file 41598_2021_93354_MOESM2_ESM.jpg]

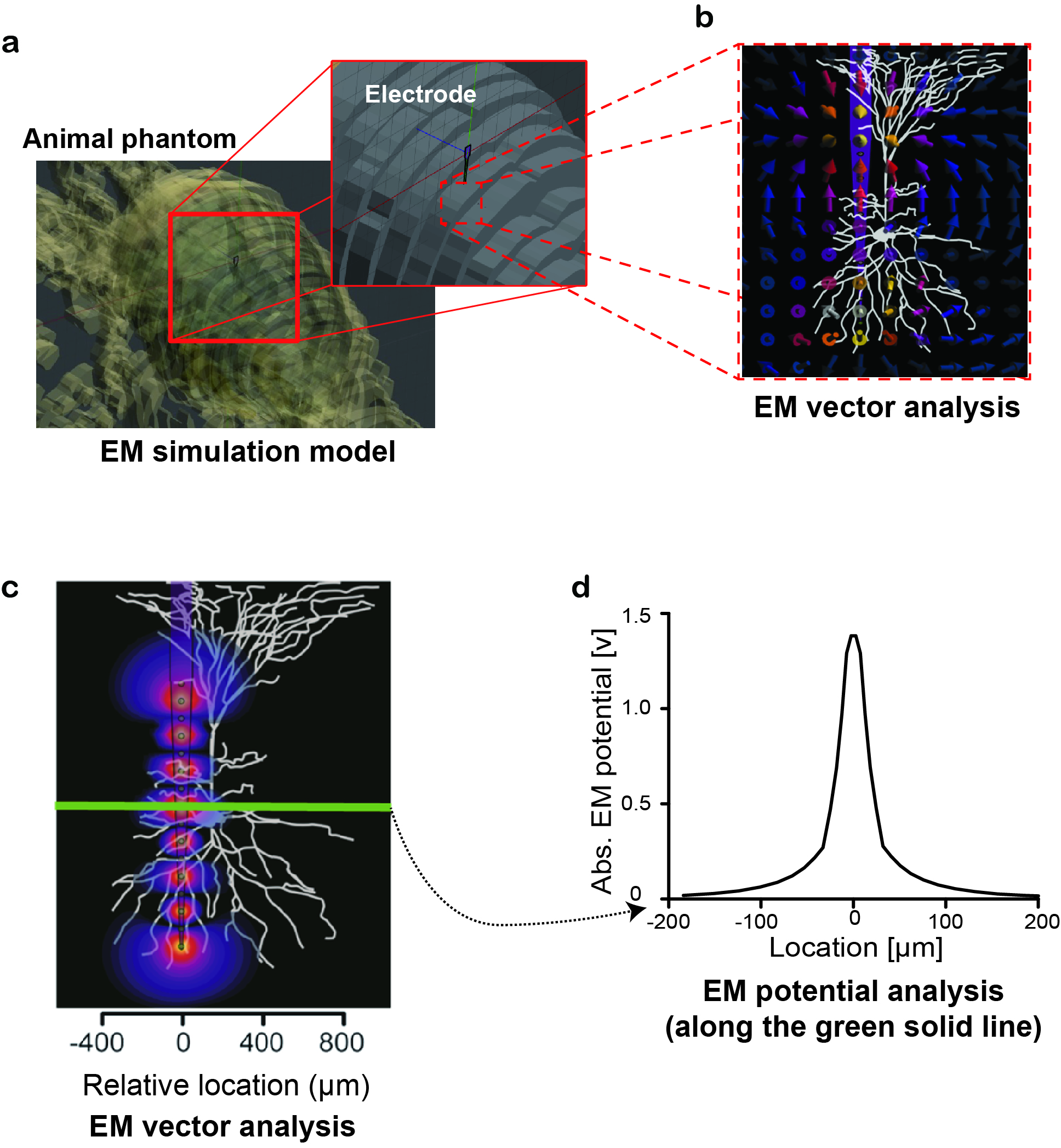

Supplement: Supplementary file 4 — Supplementary Information 4. [file 41598_2021_93354_MOESM4_ESM.jpg]
