# Supplementary material for: Subthreshold electrical stimulation as a low power electrical treatment for stroke rehabilitation
Source: Sci Rep. 2021 Jul 7;11:14048. doi: 10.1038/s41598-021-93354-x (PMC8263745; doi:10.1038/s41598-021-93354-x)
Supplement: Supplementary file 5 — Supplementary Information 5. [file 41598_2021_93354_MOESM5_ESM.docx]

**Supplementary Figures**

**Subthreshold Electrical Stimulation as A Low Power Electrical Treatment for Stroke Rehabilitation**

Kyungsoo Kim^1†^, Seung-Jun Yoo^3†^, So Yeun Kim^2.3^, Taeju Lee^4^, Sung-Ho Lim^1^, Jae-Eun Jang^5^, Minkyu Je^4^, Cheil Moon^1,2,3*^, Ji-Woong Choi^1,5*^

^1^Brain Engineering Convergence Research Center, Daegu Gyeongbuk Institute of Science and Technology, Daegu, Korea, ^2^Convergence Research Advanced Centre for Olfaction, Daegu Gyeongbuk Institute of Science and Technology, Daegu, Korea, ^3^Department of Brain & Cognitive Sciences, Daegu Gyeongbuk Institute of Science and Technology, Daegu, Korea, ^4^Department of Electrical Engineering, Korea Advanced Institute of Science & Technology, Daejeon, Korea, ^5^Department of Information & Communication Engineering, Daegu Gyeongbuk Institute of Science and Technology, Daegu, Korea

Correspondence:

*Cheil Moon, PhD, Department of Brain & Cognitive Sciences, Graduate School, Daegu Gyeongbuk Institute of Science and Technology, 333, Techno Jung-Ang Daero, Hyeonpung-Myeon, Dalseong-Gun, Daegu, 711-873, Korea. E-mail: cmoon@dgist.ac.kr; Tel: +82-53-785-1040; Fax: +82-53-785-6109

*Ji-Woong Choi, PhD, Department of Information & Communication Engineering, Graduate School, Daegu Gyeongbuk Institute of Science and Technology, 333, Techno Jung-Ang Daero, Hyeonpung-Myeon, Dalseong-Gun, Daegu, 711-873, Korea. E-mail: jwchoi@dgist.ac.kr Tel.: +82-53-785-6311; fax: +82-53-785-6311

^†^ These authors contributed equally to this work

Supplementary materials

| 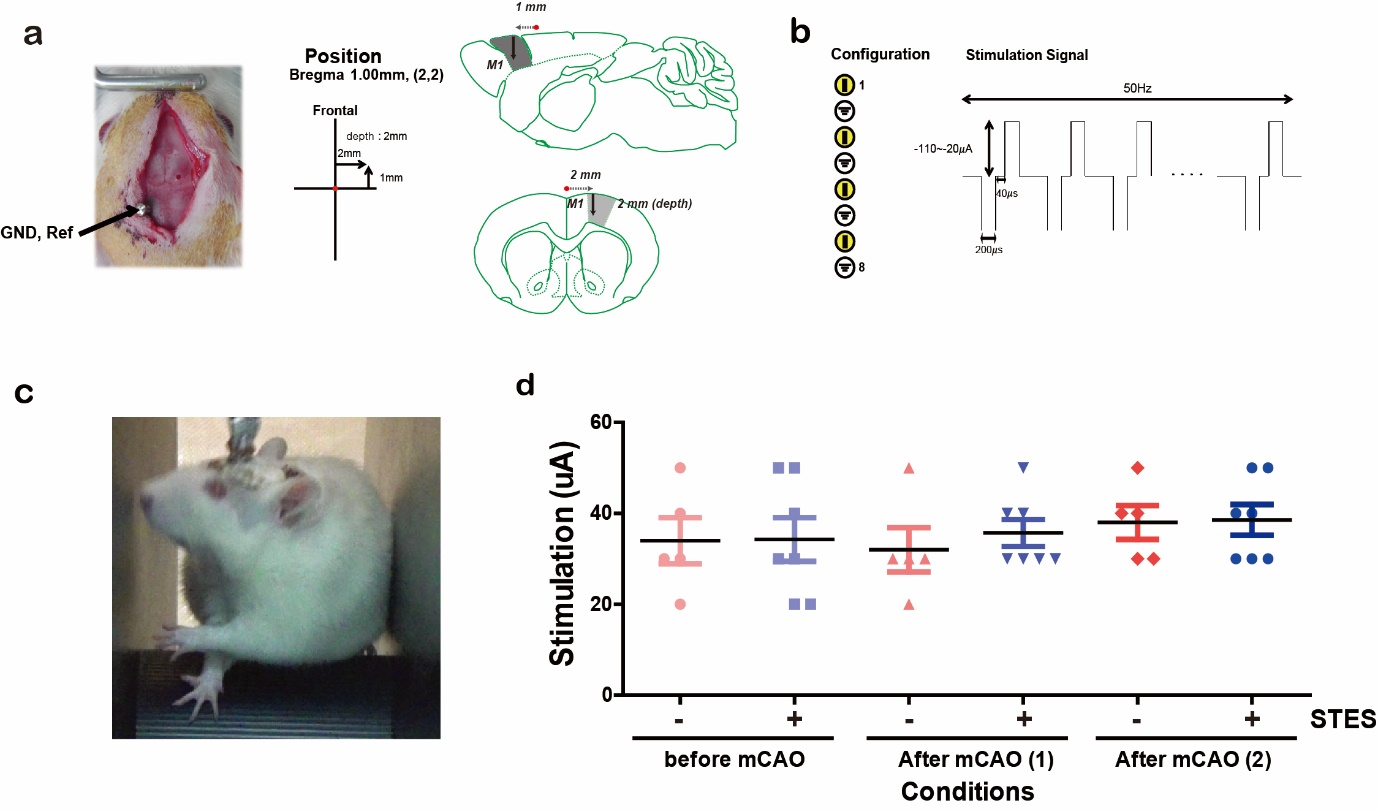 |
| --- |
| Figure S1. Electrode implantation process and confirmation of the electrical stimulation MT. (a) Position of the target site (M1) for electrode implantation. (b) Additional information on the electrode and electrical stimulation conditions are summarized. (c) Movement threshold test; the movie capture demonstrating a representative rat forelimb movement during the MT test in M1 (~40 µA). (d) Time course of movement threshold in the STES(+)/STES(-) condition. |


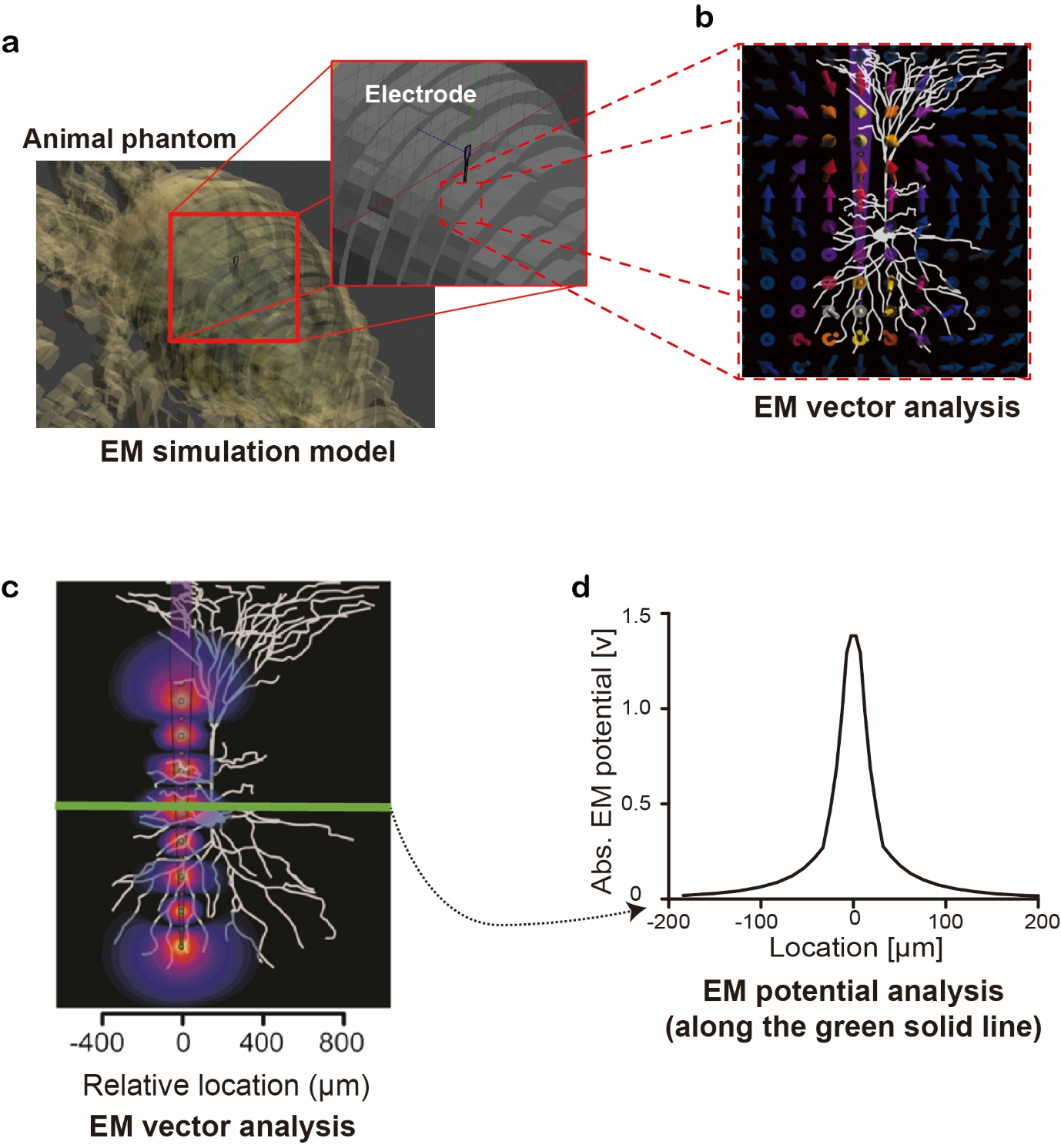


Figure S2. Electromagnetic (EM) analysis within the brain tissue using rat animal phantom simulation. (a) An electrode was implanted into the rat animal phantom. (b) Electrical current from vertically located electrode sites formed an EM field and affected nearby neurons. The 3D arrows represent EM vectors that indicate current flow. Vertically located multi-polar electrical stimulation created a vertical current stream. (c) The EM potential graph shows that electrical stimulation mostly resulted in voltage changes near electrode sites. (d) Voltage graph along with green horizontal area; 100-mV and 30-mV potential changes were observed 50 μm and 100 μm away from the electrode, respectively.
